# Predicting Cancer Risk from Germline Whole-exome Sequencing Data Using a Novel Context-based Variant Aggregation Approach

**DOI:** 10.1158/2767-9764.CRC-22-0355

**Published:** 2023-03-22

**Authors:** Zoe Guan, Colin B. Begg, Ronglai Shen

**Affiliations:** 1 Department of Epidemiology and Biostatistics, Memorial Sloan Kettering Cancer Center, New York, New York.

## Abstract

Many studies have shown that the distributions of the genomic, nucleotide, and epigenetic contexts of somatic variants in tumors are informative of cancer etiology. Recently, a new direction of research has focused on extracting signals from the contexts of germline variants and evidence has emerged that patterns defined by these factors are associated with oncogenic pathways, histologic subtypes, and prognosis. It remains an open question whether aggregating germline variants using meta-features capturing their genomic, nucleotide, and epigenetic contexts can improve cancer risk prediction. This aggregation approach can potentially increase statistical power for detecting signals from rare variants, which have been hypothesized to be a major source of the missing heritability of cancer. Using germline whole-exome sequencing data from the UK Biobank, we developed risk models for 10 cancer types using known risk variants (cancer-associated SNPs and pathogenic variants in known cancer predisposition genes) as well as models that additionally include the meta-features. The meta-features did not improve the prediction accuracy of models based on known risk variants. It is possible that expanding the approach to whole-genome sequencing can lead to gains in prediction accuracy.

**Significance:**

There is evidence that cancer is partly caused by rare genetic variants that have not yet been identified. We investigate this issue using novel statistical methods and data from the UK Biobank.

## Introduction

Different mutational processes give rise to a variety of germline and somatic variants in different genomic, nucleotide, and epigenetic contexts. In cancer genomics, extensive research has shown that the contexts of somatic variants are informative of cancer etiology and site of origin. The study of somatic mutation signatures defined by the nucleotide contexts of variants detected from whole-exome sequencing (WES) or whole-genome sequencing (WGS) has provided important insights into the processes that cause somatic alterations. Many somatic signatures have been linked to specific biological mechanisms, such as defective DNA mismatch repair, and environmental exposures, such as tobacco smoke and ultraviolet light (1–3). Moreover, it has been shown that aggregating rare somatic variants from WES or WGS based on meta-features that represent their genomic, nucleotide, and epigenetic contexts is highly effective for classifying tumor site of origin (4, 5).

The role of germline mutation contexts in carcinogenesis is less well understood. Only recently have researchers begun to make progress in identifying biological mechanisms that are responsible for the effects of germline variants (6) and in discovering germline mutation signatures associated with cancer risk (7). An analysis of over 40,000 germline whole genomes identified seven signatures, defined on the basis of the nucleotide and genomic contexts of rare variants, that are associated with biological processes such as replication timing and chromatin accessibility (6). Another study of 10,000 germline whole exomes identified seven cancer-associated signatures based on nucleotide contexts, including a signature that was enriched in cancer cases who were tobacco smokers, signatures associated with somatic mutations in oncogenes, and signatures associated with histologic subtypes and prognosis in brain, lung, kidney, and stomach cancer (7). However, further investigation is needed to confirm the relevance of germline mutation contexts to disease heritability. An open question is whether aggregating germline variants using meta-features capturing their genomic, nucleotide, and epigenetic contexts can improve cancer risk prediction. Decades of research has led to the discovery of many germline risk variants, including rare, high-risk variants in over 100 cancer susceptibility genes (8) and over 1,000 common, low-risk SNPs (9). However, known risk variants explain only a limited proportion of the heritability estimated from twin and family studies (10–14) and cancer risk models based on known risk variants have shown modest predictive power (15, 16). It has been hypothesized that missing heritability may lie in rare variants not detectable by SNP arrays (17). However, despite the increasing availability of large-scale WES and WGS data that allow for more comprehensive analyses of rare variants, these variants need to be aggregated to achieve sufficient statistical power to detect signals. Rare variant association analyses typically aggregate rare variants by gene (18), but this approach has yielded limited novel findings in WES studies of cancer (19). In the current study, we used WES data from 200,000 individuals from the UK Biobank to investigate whether aggregating rare variants using meta-features defined by mutation context can improve the performance of cancer risk models based on known risk variants. The examined meta-features include contiguous regions that span the entire genome and capture genomic and epigenetic contexts, as well as categories of single base substitutions defined by the type of base change and the flanking nucleotides. Since heritability analyses and risk prediction modeling for cancer have largely focused on SNPs, we also applied the aggregation approach to SNP array data from 500,000 individuals from the UK Biobank.

## Materials and Methods

### UK Biobank

The UK Biobank (RRID:SCR_012815) is a population-based study of over 500,000 individuals from the United Kingdom that is 94% White (20). Participants aged 40–69 were recruited from 2006 to 2010. They provided detailed information on sociodemographic, health, and lifestyle factors, as well as biological samples. Cancer cases were identified through linkage to national cancer registries established in the 1970s. We used the second release of WES data from the UK Biobank, which included 200,619 participants (21). For WES, exomes were captured with the IDT xGen Exome Research Panel v1.0 and sequenced using Illumina NovaSeq 6000 (>20× coverage at 95% of targeted bases); variant calling was performed using GATK 3.0 (RRID:SCR_001876). Separately we analyzed SNP array data (22) for 438,242 participants sequenced by the UK Biobank Axiom Array, containing approximately 850,000 markers (samples from the first release of genome-wide genotyping data were sequenced using a different array, UK BiLEVE, and were excluded from our analyses to minimize confounding due to technical differences). We also used imputed SNP data (version 3), which increased the number of available SNPs to 96 million.

### Cases and Controls

To reduce confounding due to population stratification, we restricted our analyses to participants of European ancestry (data field 22006), that is, those who self-identified as “White British” (data field 21000) and were inferred to be of European ancestry based on a principal components analysis of SNP data. Participants with at least one prevalent or incident diagnosis of a malignant cancer based on ICD-9 (data field 40013) or ICD-10 (data field 40006) codes were defined as cases (participants with only *in situ* diagnoses were not included). Those with multiple diagnoses were defined as a case for their first cancer. We focused on the nine most common cancer types—breast cancer, colorectal cancer, endometrial cancer, kidney cancer, lung cancer, melanoma, non–Hodgkin lymphoma, ovarian cancer, prostate cancer—and testicular cancer, for which high SNP heritability (heritability explained by all genotyped SNPs) was reported in a previous UK Biobank study (13). Because a family history of cancer can indicate a strong germline influence, we also considered restricting the cases to those with a first-degree family history of the same cancer (data fields 20110, 20107, 20111; family history information was only available for breast, colon, lung, and prostate cancer). We conducted this restricted analysis for breast and prostate cancer because these were the only cancer types that had a sufficient number of cases with a family history. For each cancer type, we sampled controls from the set of participants who never had a cancer diagnosis (malignant or *in situ*). We used 1:1 matching on birth year and sex, with the requirement that the control was alive at the age of diagnosis of the case. The sample sizes for the cancers of interest are provided in Table 1.

**TABLE 1:** Number of cases by cancer type

Cancer site	No. of cases with WES data	No. of cases with SNP data
Breast	4,722	11,902
Breast (Family history)	569	1,362
Colorectal	1,663	4,640
Endometrial	557	1,485
Kidney	382	1,125
Lung	625	2,054
Melanoma	1,282	3,269
Non–Hodgkin lymphoma	739	1,794
Ovarian	398	1,052
Prostate	2,970	8,700
Prostate (Family history)	449	1,236
Testicular	253	627

### Variant Filtering

Using the same quality control procedure as in a previous WES UK Biobank study (21), we analyzed WES variants from autosomal chromosomes that satisfied the following criteria: variant missingness <10%, Hardy Weinberg equilibrium *P* value > 1e-15, minimum read coverage depth of 7 for SNVs and 10 for indels, and at least one sample per site passed the allele balance threshold 0.15 for SNVs and 0.20 for indels. For the SNP array data, we included SNPs from autosomal chromosomes that had a call rate >95% and Hardy Weinberg equilibrium *P* value > 1e-5, as in a previous study using the UK Biobank SNP array data (13). For the imputed SNPs, we used those with imputation quality score ≥ 0.8. We used PLINK v1.9 (RRID:SCR_001757; ref. 23) to filter the genotyped and imputed SNPs.

### Definition of Meta-features

Chakraborty and colleagues developed a context-dependent learning approach that aggregates rare somatic variants using predefined meta-features that capture their genomic and nucleotide contexts, achieving dimension reduction and signal condensation (4, 5). We adapted this approach to germline genotypes, developing risk models using meta-features constructed from WES data as well as risk models using meta-features constructed from SNP array data.

A meta-feature can be interpreted as a variant annotation (e.g., membership in a given gene). Suppose there are *d* variants (they can be genotyped using any technique) and *p* meta-features. Let 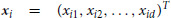 be the genotype vector for individual *i*, where *x*_*ij*_ is the allele count for variant *j*. Let 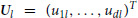 contain the values of meta-feature *l* for all variants *{j}*. For example, if meta-feature*l* corresponds to membership in *BRCA1*, then *u*_*jl*_ indicates whether variant *j* is in *BRCA1*. Then 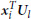 is the variant burden associated with meta-feature *l* for individual *i* and the set of predictors 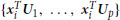 can be used as model inputs.

We considered three categories of meta-features that describe the genomic, nucleotide, and epigenetic contexts of variants: (i) 114 known moderate/high-risk cancer susceptibility genes (8), (ii) 2,734 contiguous 1 Megabase (Mb) chromosomal windows, and (iii) 96 single base substitution (SBS-96) categories. These meta-features were modeled as counts and were computed using the hidgenclassifier R package. In computing them, we restricted to rare variants with minor allele frequency (MAF) <1% in the entire UK Biobank cohort. Meta-features with counts <5 in the training set were not included in the models. When counting the number of variants in a cancer susceptibility gene, we restricted to variants annotated as loss-of-function by the Ensembl Variant Effect Predictor (RRID:SCR_007931; ref. 24) or annotated as pathogenic or likely pathogenic by ClinVar (RRID:SCR_006169; ref. 25). The cancer susceptibility genes are listed in Supplementary Table S1. They were only used in the WES models, that is, they were excluded from models that investigated SNP array data. The contiguous 1 Mb regions span the entire genome, serving as a form of region-based aggregation. Epigenetic influences drive differences in mutation rates across genomic regions (6), so the windows reflect both genomic and epigenetic contexts. SBS-96 categories represent the 96 possible trinucleotide contexts for single base substitutions, with each context consisting of a substitution (C>A, C>G, C>T, T>A, T>C, or T>G) and its two flanking nucleotides. These contexts have been used to define somatic mutation signatures that reflect etiologically distinct cancer subtypes (2). Recent work has also shown that there are germline signatures based on nucleotide context that are associated with cancer risk (7).

In our prediction models, we also used cancer-associated SNPs and polygenic risk scores (PRS) obtained from published genome-wide association studies (GWAS). For the WES models, we used individual cancer-risk SNPs from the NHGRI-EBI GWAS Catalog (RRID:SCR_012745; ref. 9). For each cancer type, we selected SNPs that passed the genome-wide significance threshold in a published GWAS for the given cancer. For the SNP models, we used a previously published PRS from the PGS Catalog (26) for each cancer type (27–33) (we did not use PRSs in the WES models because most of the SNPs included in PRSs are not located in coding regions). The SNPs included in the WES models are provided in Supplementary Table S1. The PRSs used in the SNP models are provided in Supplementary Table S2.

### Model Training and Validation

For each cancer type and each data type (WES data and SNP array data), we developed two logistic regression models, a benchmark model based on known risk variants (cancer-associated SNPs and, in the case of WES data, cancer susceptibility genes) and an expanded model based on known risk variants, 1 Mb windows, and SBS-96 categories. In the expanded models, Lasso regularization was applied to the 1 Mb windows and SBS-96 categories but not to the known risk variants. To adjust for confounding by population structure, we “residualized” the predictors by subtracting the effects of the top five principal components (PC) computed from linkage disequilibrium (LD)-pruned common SNPs (34). In the WES analyses, a set of 19,001 LD-pruned SNPs with MAF >1% was obtained by pruning those with a pairwise *r*^2^ > 0.5 within a 1,000 kb window. Pairwise *r*^2^ values were obtained from the 1000 Genomes Project (RRID:SCR_008801) using the LDLinkR R package. In the SNP array analyses, we used genetic PCs previously computed by the UK Biobank (data field 22009). Model performance was evaluated by computing the average area under the receiver operating characteristic curve (AUC) across five iterations of 5-fold cross-validation. We also performed Wald tests for each of the individual predictors in the models, adjusting for the top five PCs.

### Power Calculations for WES Association Tests

**FIGURE 1:**
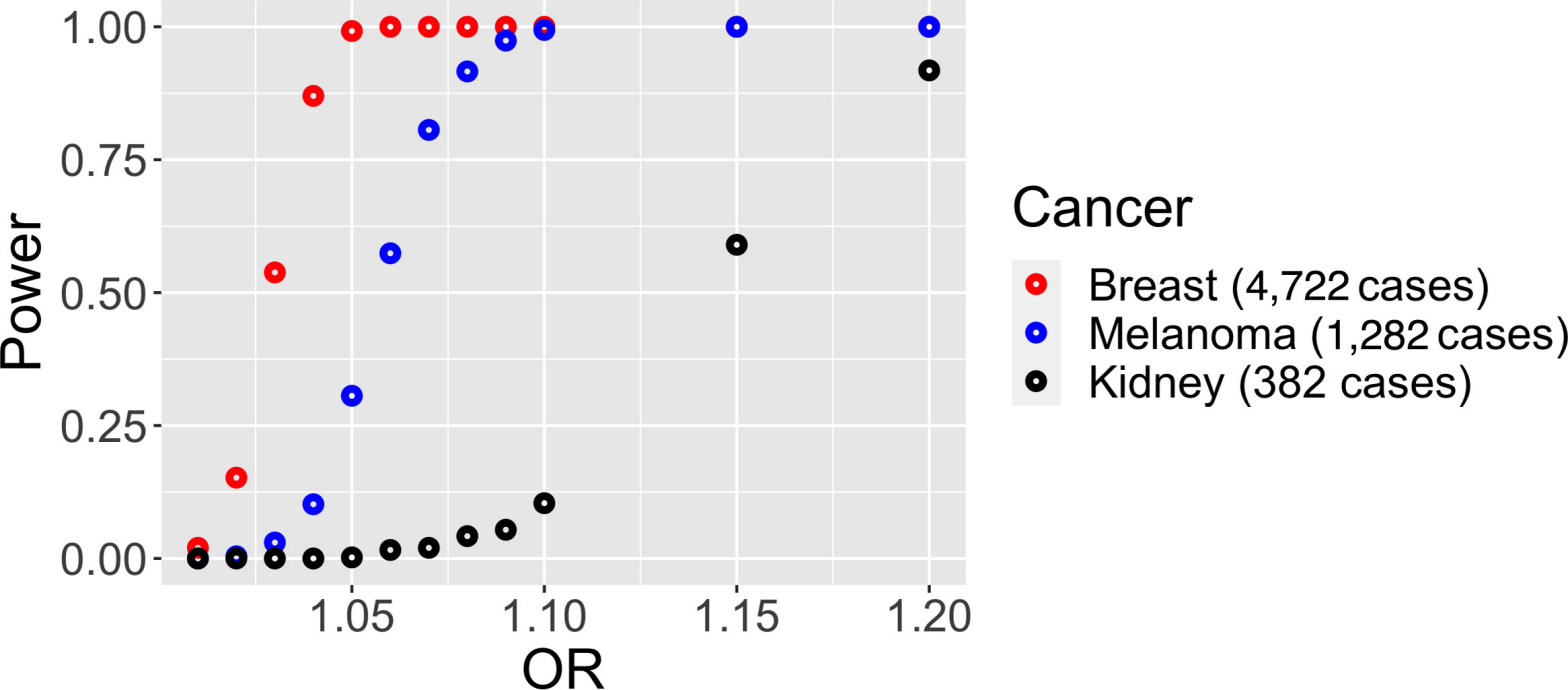
Simulation-based power for WES association tests. For breast cancer, melanoma, and kidney cancer, we estimated the power of performing a Wald test for the SBS-96 category consisting of G[T>C]T substitutions using simulations where we varied the OR for this SBS-96 category.

Simulation-based power estimates for association tests for selected cancer types (breast cancer, which had the largest sample size, melanoma, and kidney cancer) based on WES data are provided in Fig. 1. We focused on estimating the power of performing a Wald test for the SBS-96 category consisting of G[T>C]T substitutions (T to C substitutions flanked by G and T) because the mean count for this category was close to the mean count across all SBS-96 categories in the UK Biobank. For various ORs and numbers of cases *n* (corresponding to the sample sizes for the selected cancer types in the WES analysis), we performed 500 iterations of the following procedure, then estimated the power as the proportion of iterations resulting in a significant *P* value for the meta-feature (after Bonferroni adjustment): (i) for each individual in the WES analysis, generate their case–control status *Y* based on the logistic regression model 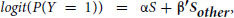 where α is the log OR, *S* is the individual's count, β is the vector of log ORs for the predictors besides the SBS-96 category of interest, and ***S***_***other***_ is the vector of the individual's counts for those meta-features (specifically, *Y* is sampled from a Bernoulli distribution with probability 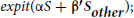 (ii) sample *n* cases and *n* controls and perform a Wald test for the association between the SBS-96 category and cancer status. For the meta-features, we used the actual counts from the UK Biobank WES data. β was fixed across all iterations: the effect size for each meta-feature in ***S***_***other***_ was obtained by fitting a univariate logistic regression for the meta-feature and the cancer of interest using the actual UK Biobank data. As seen in Fig. 1, the power was very high for breast cancer and melanoma even for modest effect sizes (e.g., ORs of around 1.1).

### Ethics Approval and Consent to Participate

This study was performed using data from the UK Biobank under application 74025. The research was performed in accordance with the principles of the Helsinki Declaration.

### Data Availability

The data that support the findings of this study are available from the UK Biobank and are accessible to approved researchers. Our analyses were conducted under application 74025.

## Results

The cross-validation AUCs of the risk models are shown in Table 2. The WES models had low discrimination (we did not train WES models for testicular cancer because no known risk variants were available in the WES data). The highest AUC was 0.60, which was achieved by the models for melanoma. The expanded models did not show improvements in AUC over the benchmark models. We also conducted a sensitivity analysis where we included missense variants instead of restricting to loss-of-function variants for the known cancer genes (Supplementary Table S3), because missense variants can also have strongly deleterious effects. However, the models with missense variants did not perform any better than the models with loss-of-function variants.

**TABLE 2:** Average model AUCs across five iterations of 5-fold cross-validation

	AUC			
	WES model		SNP model	
Cancer site	Benchmark	Expanded	Benchmark	Expanded
Breast	0.55	0.55	0.61	0.61
Breast (Family history)	0.55	0.55	0.63	0.63
Colorectal	0.53	0.53	0.58	0.58
Endometrial	0.52	0.51	0.57	0.57
Kidney	0.49	0.50	0.56	0.56
Lung	0.52	0.52	0.54	0.54
Melanoma	0.60	0.60	0.62	0.62
Non–Hodgkin lymphoma	0.48	0.48	0.54	0.54
Ovarian	0.53	0.53	0.55	0.55
Prostate	0.56	0.56	0.66	0.66
Prostate (Family history)	0.58	0.58	0.69	0.69
Testicular	–	–	0.68	0.68

NOTE: Benchmark WES model: model based on known risk variants from WES data. Expanded WES model: model based on known risk variants and meta-features from WES data. Benchmark SNP model: model based on known risk variants from SNP array data. Expanded SNP model: model based on known risk variants and meta-features from SNP array data. We did not fit WES models for testicular cancer because no known risk variants for testicular cancer were available in the WES data.

The SNP models had higher AUCs than the corresponding WES models for all cancer types. The SNP models with the highest AUCs were the ones for prostate cancer (AUC = 0.69 for the models for prostate cancer with family history and AUC = 0.66 for the models for prostate cancer with or without family history) and testicular cancer (AUC = 0.68). For all cancer types, the benchmark SNP model and expanded SNP model had the same AUC.

Significant results from association tests for the individual predictors in the models are provided in Supplementary Tables S4 and S5. In the WES analyses, four significant associations were found (Supplementary Table S4): in addition to three known associations (one gene and two GWAS SNPs), one SBS-96 category was significantly associated with melanoma (T[C>A]G). In the SNP analyses, all of the significant predictors were PRSs. Every PRS was significant except for the one for ovarian cancer (Supplementary Table S5).

## Discussion

Two previous studies have used SNP array data from the UK Biobank to estimate the SNP heritability of various cancer types (12, 13). Rashkin and colleagues (13) looked at 18 common cancer types, including the ones in our study. Among the cancer types in our study, Rashkin and colleagues’ heritability estimates ranged from 0.07 for colon and ovarian cancer to 0.26 for testicular cancer. Jiang and colleagues (12) looked at six cancer types (breast, colon, lung, oral, ovarian, pancreatic, and prostate) and obtained heritability estimates ranging from 0.03 for ovarian cancer to 0.18 for prostate cancer. While we did not estimate heritability directly, heuristically, our SNP models for prostate cancer and testicular cancer had the highest AUCs and our SNP models for ovarian cancer had AUCs that were among the lowest, which is consistent with prostate and testicular cancer being one of the most heritable and ovarian cancer one of the least heritable cancers in the two previous studies. Overall, the AUCs of our SNP models are similar to previously reported AUCs of PRSs (15, 35–37).

Previous WES studies of cancer have mainly focused on pathogenic variants in a small set of known and candidate cancer susceptibility genes (38, 39). Exome-wide association studies have also been conducted, but they have been hampered by low statistical power and have identified few novel genetic risk factors (19, 40). Several studies have used UK Biobank WES data to perform rare variant association analyses where various types of cancer were included as phenotypes of interest (41–44). However, in these studies, significant results for cancer phenotypes were restricted to known cancer susceptibility genes. In this study, we sought to address the fundamental question of whether summarizing genome-wide germline mutation contexts can help explain the heritability of complex traits. This was the first study to investigate whether aggregating rare germline variants from WES or SNP array genotyping based on their genomic, nucleotide, and epigenetic contexts can improve the performance of cancer risk models. Motivated by Chakraborty and colleagues’ somatic WES and WGS analyses showing that these contexts are informative of tumor site of origin (4, 5) and Xu and colleagues’ germline WES study showing associations between these contexts and cancer risk (7), we applied context-based aggregation in a larger germline WES dataset to develop cancer risk models. However, in contrast to Xu and colleagues’ study, we did not find evidence that the meta-features contribute to cancer risk. For most of the cancer types considered, adding meta-features grouping rare WES variants to a benchmark model with known exonic risk variants did not improve performance, indicating that the meta-features may contain more noise than signal. Adding meta-features grouping rare SNPs did not improve the performance of the SNP benchmark model for any cancer types. In the WES association analyses, one SBS-96 category was significantly associated with melanoma, but further study is needed to confirm this association.

Overall, the AUCs for the WES models were low, ranging from 0.48 to 0.60, suggesting that heritability is not mainly driven by exonic variants. It is possible that larger sample sizes and/or alternative variant aggregation methods are needed to detect signals from the exome, but the relatively small contribution of the exome to heritability is further supported by the higher AUCs of the SNP models, ranging from 0.54 to 0.69. SNP arrays capture genome-wide SNPs, the vast majority of which are beyond the exome. However, SNP arrays provide relatively little information on rare variants, even when combined with imputation. WGS is needed to study genome-wide rare variants and may be essential for identifying sources of missing heritability and achieving more substantial gains in prediction accuracy. In the somatic setting, it has been shown that WGS is much more informative than WES for classifying tumor site of origin (5). In the germline setting, a recent heritability study of height showed that a considerable portion of the missing heritability for this trait lies in rare variants from WGS (45). WGS can potentially also uncover much of the missing heritability of cancer. The UK Biobank has recently released WGS data for 200,000 participants, providing an opportunity to investigate this hypothesis. Other possible explanations for missing heritability include gene–gene and gene–environment interactions and structural variation. Traditional models of heritability assume that there are no gene–gene or gene–environment interactions, but there is evidence that this assumption is unrealistic and may lead to inflated estimates of total heritability (46, 47). Another possible source of missing heritability is structural variation (17), which is not well captured by short-read WGS. Little is known about the contribution of structural variants to cancer risk, and this is a new direction to explore as sequencing technology continues to advance.

## Conclusions

In summary, we used germline WES and SNP array data from the UK Biobank to develop risk models for 10 cancer types based on known risk variants and meta-features representing mutation context. Despite previous evidence that these meta-features are associated with cancer risk, they did not add predictive value beyond known risk variants. The WES models had lower AUCs than the SNP models, suggesting that exonic variants contribute relatively little to the heritability of common cancer types. It is possible that expanding to WGS can lead to gains in prediction accuracy.

## Supplementary Material

Table S1 Table S1: Known risk genes (from Rahman 2014) and SNPs (from NHGRI-EBI GWAS Catalog) in WES models.

Table S2 Table S2: Polygenic risk scores used in SNP models (obtained from the Polygenic Score Catalog).

Table S3 Table S3: AUCs of benchmark WES models using loss-of-function and missense variants.

Table S4 Table S4: Significant predictors from individual association analyses based on WES data. The significance threshold for each cancer type was 0.05/n, where n is the number of predictors tested for the cancer type (ranging from 2541 for kidney to 2596 for breast).

Table S5 Table S5: Significant predictors from individual association analyses based on SNP array data. The significance threshold for each cancer type was 0.05/2824 (2824 predictors were tested for each cancer type).
